# Quantitative Analysis of Cell Nucleus Organisation

**DOI:** 10.1371/journal.pcbi.0030138

**Published:** 2007-07-27

**Authors:** Carol Shiels, Niall M Adams, Suhail A Islam, David A Stephens, Paul S Freemont

**Affiliations:** National Center for Biotechnology Information, United States of America

## Abstract

There are almost 1,300 entries for higher eukaryotes in the Nuclear Protein Database. The proteins' subcellular distribution patterns within interphase nuclei can be complex, ranging from diffuse to punctate or microspeckled, yet they all work together in a coordinated and controlled manner within the three-dimensional confines of the nuclear volume. In this review we describe recent advances in the use of quantitative methods to understand nuclear spatial organisation and discuss some of the practical applications resulting from this work.

## Introduction

**Figure 1:**
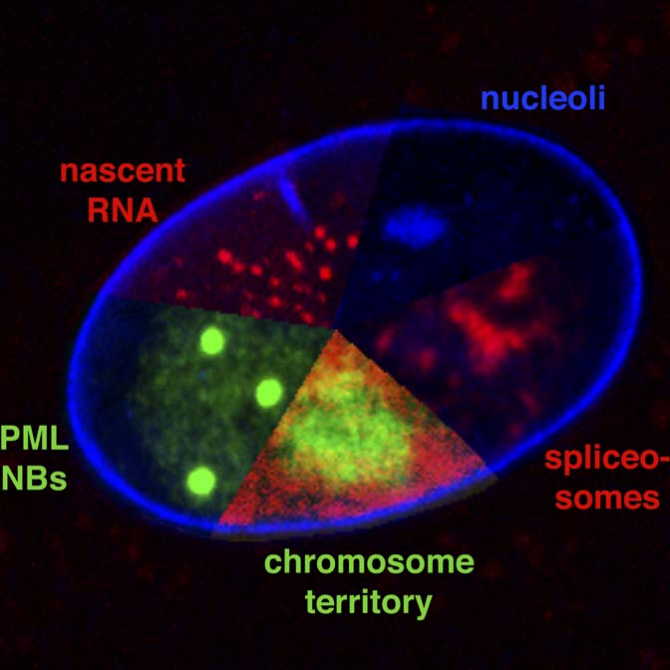
The Mammalian Interphase Nucleus The diversity in size and number of some of the major functional nuclear compartments is shown. PML NBs, PML nuclear bodies.

The non-uniform nature of the nucleus has been obvious since the first early studies describing the arrangement of satellite DNA sequences in interphase nuclei [1,2]. Advances in the resolution with which we can now observe the interphase nucleus have resulted in a wealth of cell biology data and have allowed the distribution patterns of many nuclear proteins to be observed and catalogued (some examples are shown in Figure 1). However, the rules governing the structure and internal organisation of the nucleus still remain elusive. The interior of the cell nucleus is made up of a dynamic mix of membraneless compartments of varying functional capacity. Whether there is structure and reason to this organelle in the form of an organising nuclear matrix, itself a highly contentious structure [3], or whether there is self-organisation [4], perhaps governed only by the laws of molecular crowding [5], still remains to be established.

For an organelle as complex as the cell nucleus, the questions asked about its organisation are surprisingly simple. One of the most obvious is how each of the nuclear compartments is distributed in the nuclear space. A random distribution, with the inability to predict where a particular compartment may be located, might be interpreted to imply a lack of order or organisation to the nuclear interior, since no region of the nucleus is any more, or any less, likely to accommodate the compartment of interest. Conversely, if compartments are either more regular or clustered in their distribution, this would suggest that there are some, as yet unknown, mechanisms responsible for their organisation. A second question concerns the interrelationships between nuclear compartments. For example, do certain compartments tend to associate, and, if so, is it a statistically significant association having functional and/or organisational implications? These simple questions are deceptively difficult to answer, but in the expanding era of quantitative biology, a number of old and new approaches are being applied to nuclear organisation in an attempt to understand the underlying spatial relationships.

## Imaging Compartments Inside the Cell Nucleus

Quantitative analysis of the nuclear interior relies on the ability to visualise compartments within this space with a high degree of accuracy, with the quality of input data greatly influencing the confidence with which quantitative analysis can be interpreted. Fluorescent imaging methods, combined with either confocal or wide-field microscopy, are most commonly used for collection of two- or three-dimensional (2D or 3D) images of the cell nucleus. Ideally, minimal cell preparation methods should be used in order to preserve the native state existing in live cells. Visualisation of endogenous nuclear proteins by immunofluorescence requires the fewest cell preparation steps, although it does involve cell fixation and permeabilization, so some degree of disturbance must be tolerated. Also, certain nuclear compartments are more sensitive than others to the methods used; for example, the immunostaining of speckles and Cajal bodies is similar for a range of fixation protocols, whereas RNA polymerase II transcription foci are extremely sensitive and a proportion may be extracted from their usual nuclear locations under suboptimal fixation conditions [6]. Visualisation of specific chromosomes or genes within the chromatin compartment requires more aggressive cell preparation methods, and the type of method employed has significant consequences for quantitative measurements. If DNA alone is being studied, for example, when studying the nuclear location of chromosome territories by fluorescence in situ hybridisation (FISH), it is common to employ a hypotonic treatment in combination with methanol/acetic acid fixation. This treatment leads to an increase in contact between the cell nucleus and its support, resulting in a substantial increase in nuclear diameter and nucleus flattening [7], such that only 2D analysis can be performed on the resulting cell preparations. Also, the chromatin does not move outward uniformly in proportion to this increase in nuclear diameter, resulting in a radial distortion of gene topology [7]. When both nuclear proteins and specific DNA sequences are being studied, a compromise must be reached in the cell preparation method such that it permeablizes the cell sufficiently for the DNA to be detected, yet at the same time is mild enough that the nuclear proteins are not extracted. The technique used in this instance, immuno-FISH, generally employs brief prefixation permeabilization steps accompanied by mild paraformaldehyde-based fixation. Cells prepared by this method do not have such major changes in nuclear chromatin architecture compared with those prepared for standard FISH, but it has been demonstrated that the heat denaturation step required to visualise the DNA still destroys details at the ultrastructural level [8].

Another important factor may be the degree of variability in the quality of images captured by either wide-field or confocal microscopy and whether images are deconvolved prior to analysis, since the more manipulations data undergo before analysis, the more variability and inconsistency there is likely to be when comparing results between different laboratories. Also, in the recent past, the computer power required to process 3D data from a large number of nuclei was often a limiting factor. In some cases, the information from a 3D data stack was converted back to 2D data for the purposes of analysis. Alternatively, a single 2D image though the midpoint of the nucleus was used instead of the full 3D image. This is probably acceptable for distribution studies when the number of nuclear compartments to be studied is large, such as in the case of double minute chromosomes [9], but may be problematic if the nuclear compartment under investigation is restricted to only a small subsection of the nucleus. In these cases, the depth of field of the objective (i.e., the vertical distance from which information can be captured) may be important [10]. When associations between nucleus compartments are of interest, it has been argued by Jacobs and colleagues that constraining the analysis to two dimensions actually makes statistical tests more stringent, since random (expected) colocalisations will be less frequent if the foci are given more room to roam, and will lower the threshold of the significance [10]. Otherwise, meaningful results have been obtained by studying distribution patterns in flattened 2D cell nuclei [11,12]. This method cannot be used to calculate absolute distances because of the disruptive effects of the cell preparation method on cell nucleus topology, but is generally considered sufficient for statistical analysis of relative distance measurements. There are some instances where the importance of considering the full 3D image can be convincingly demonstrated. For example, when examining the X chromosome territory, single 2D optical sections consistently showed a smaller diameter for the inactive X than for its active counterpart, suggesting a smaller inactive X territory volume. A closer inspection of the 3D data, however, revealed that the inactive X territory was present in a greater number of vertical sections than the considerably flatter active X territory, and that in fact the two X territories were not dissimilar in volume [13].

**Figure 2:**
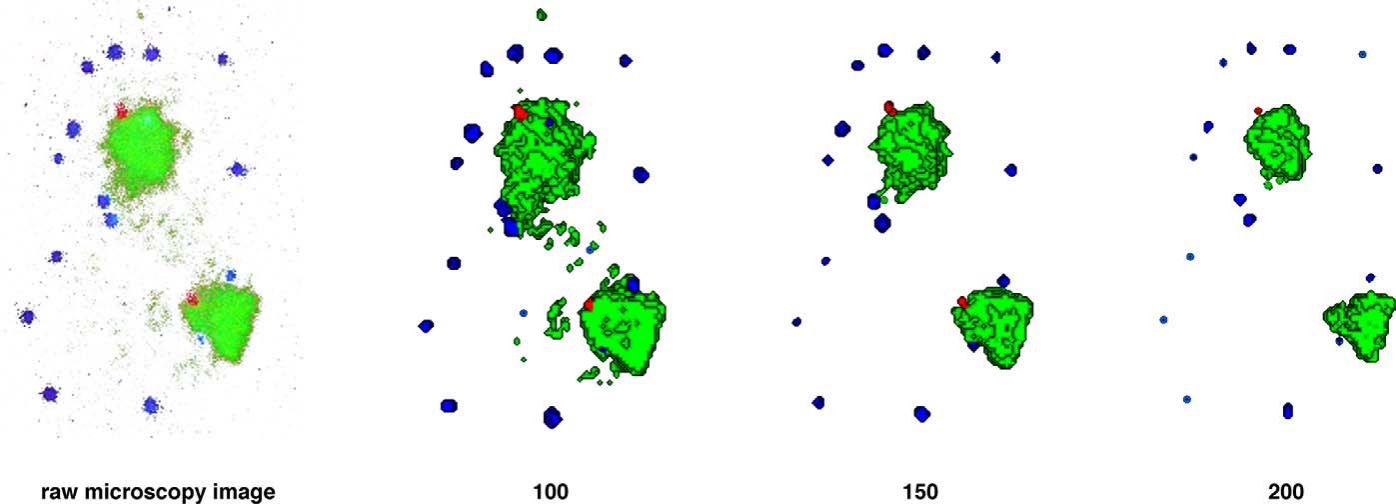
Segmentation of Cell Nuclei Nuclear compartments imaged by fluorescence microscopy often have indistinct outlines, and image segmentation methods must be implemented to subtract background from genuine signal. In this example, chromosome territories (green), PML nuclear bodies (blue), and genomic loci (red) imaged in primary human fibroblast cell nucleus (leftmost panel) have been segmented at a range of thresholding values: 100, 150, and 200 as indicated. As the threshold value increases, the proportion of signal designated as background increases, and this influences both the number of compartments and their respective compartment volume.

One drawback of studying the nucleus by indirect means is that the resulting image is not necessarily a true representation of the actual nuclear compartment. The efficiency of immunolocalisation of antibodies at nuclear compartments, which can be affected by factors such as avidity, suitability, and titre, can greatly influence the fluorescence intensity profile and therefore the overall size of the nuclear compartment. Compartments visualised using immunocolocalisation in this manner generally have indistinct borders, with the fluorescence intensity profile tailing off gradually. Many quantitative methods, however, require image segmentation or thresholding to convert the raw data into a set of manageable 3D coordinates. Segmentation methods effectively define a boundary between the nuclear compartment of interest and the remainder of the nucleus. Thresholding can have a profound effect on the volume of the resulting compartment, for example, the mean volume of centromeres in sperm nuclei can vary by almost a factor of three depending on the thresholding value chosen [14]. In Figure 2, the effect of different thresholding values on the size of compartments is clearly demonstrated. The value chosen can also influence colocalisation studies between different nuclear compartments; too low a threshold may result in background pixels being included in the analysis [15], while too high a threshold may lead to low-intensity signal being ignored [16]. To avoid bias due to user-defined thresholding values, it is common to use a range of reasonable thresholds followed by averaging [13]. A more objective approach may be to instead choose a threshold based on total signal above background; this can easily be done by measuring signal intensity from those regions of the image where the nuclear compartment is excluded, the value of which acts as the mean noise level [16]. Getting the balance right between exclusion of noise pixels and inclusion of real but lower intensity data pixels will be vitally important for any subsequent quantitative analysis to give meaningful results. It also underscores the importance of considering the full dynamic range of the fluorescent image before drawing conclusions regarding the distribution of nuclear compartments [16]. In many respects it would be preferable to avoid segmentation methods altogether. Defining boundaries around compartments may in fact create an artificial reality: not only is there likely to be a halo effect from the use of fluorescent antibodies that will most likely lead to an overestimation of nuclear compartment size, but there is also the consideration that nuclear compartments are not enclosed by internal membranes and a definitive border may be based on an erroneous concept, although electron microscopy methods have provided evidence for boundaries of identifiable structures such as promyelocytic leukaemia (PML) bodies and nucleoli.

One final, and perhaps most important, factor for accurate quantitative analysis is the source of input data, in other words, the type of cell chosen for analysis. To date, much of our knowledge of cell nucleus organisation is based on studies of cells grown in culture, rather than cells from specific tissues. One common compromise is to use freshly isolated and minimally cultured primary cell populations, and although this is far more desirable than the use of abnormal cell lines derived from cancerous tissues, it may still be far from ideal. The effects of changes in the external environment on the cell nucleus, such as that encountered when a cell is transferred from a specific 3D tissue environment to a 2D cell culture system, have not been adequately studied to date. The finding that reconstitution of the basement membrane can have significant effects on the location of a number of important nuclear proteins [17] points to an important relationship between nuclear organisation and the surrounding tissue environment. Currently it is uncommon and technically challenging to study cells in sections from tissues, but it may well be shown to give the most meaningful and relevant results in the future. In addition, the majority of studies have so far been conducted on static, fixed cells. In the future, live cell imaging of fluorescent fusion proteins is likely to become more commonplace and is the only suitable method available to advance our understanding of the real-time interactions occurring between nuclear compartments.

## Nuclear Positions and Preferences

It is still unclear to what extent nuclear structure is linked to nuclear activity and in particular whether major changes in nuclear architecture are correlated with changes in cell state. This is one of the main reasons why the nuclear positioning of compartments such as centromeres, telomeres, genes, and chromosome territories has been studied so intensely. A major stumbling block, however, has been the absence of common spatial reference points between cells and thus the inability to align cells in the same orientation when comparing organisation across cells or cell types. To date there is no reported cell organelle or nuclear marker that can perform this function, although the centrosome [18] and the X chromosome [19] have both been considered. It is possible that some types of cell nuclei, especially those in culture, do not possess such polarisations or that we currently lack markers to recognise them [20].

**Figure 3:**
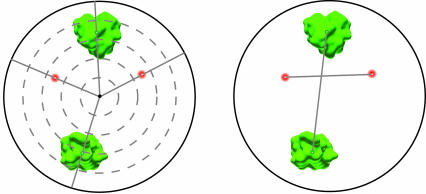
Describing Spatial Locations in the Nucleus: Radial versus Relative Radial methods (left panel) use the nuclear centre as a reference point when describing the spatial positioning of nuclear compartments. The nucleus is either divided into concentric shells of equal volume (or equal area if the analysis is in 2D), shown here as five concentric shells, and the proportion of fluorescent signal in each shell is used to quantify radial position. Otherwise, the distance between the compartment centre and the nucleus centre is expressed as a fraction of the nuclear diameter. For those compartments of irregular outline, such as the chromosome territories shown here, the intensity gravity centre is often used to approximate territory position. An alternative method is to use relative spatial positioning of compartments using either the normalised distance between compartment centres (right panel) or the orbital angle formed by a line connecting compartment centres and the nucleus centre (left panel).

A common solution is to use some form of radial analysis in which the nuclear centre is used as the reference point and the remainder of the nucleus is subdivided into concentric rings or shells from the centre to the periphery (Figure 3). The positioning of nuclear compartments relative to the geometric centre is then calculated and usually expressed as a normalised distance because of variation in nuclear size and shape within cell populations. Also known as erosion analysis or nuclear peeling, this method allows a fast evaluation of nuclear positioning and is ideally suited for investigating large sample numbers. Early studies quickly demonstrated that there is a remarkable degree of variability in the spatial positioning of nuclear compartments within a cell population, but that positioning preferences could be detected by looking for non-uniform patterns using statistical means. Often this is done by comparing the nuclear distribution of compartments to look for significant differences or trends in radial positioning [21–25]. Alternatively, the observed experimental distribution is compared with theoretical models of distribution calculated either analytically or by computer simulation [26–31]. Deviation from uniformity suggests there is a preference for compartments to be located at a specific nuclear location, for example, the nuclear interior may be preferred. One recent variation on radial analysis has been to fit the smallest possible convex set of polygons around the data of interest. The level of flatness of the resulting ellipsoids can be assessed based on the ratio of the ellipsoid radii. For example, when this analysis was applied to telomeres in primary mouse lymphocytes, their distribution was found to change from a spherical-like pattern during G1 to a flattened telomeric disk during the G2 phase [32].

A second solution to the lack of reference points in the nucleus is to use relative spatial positioning (Figure 3). One example of this utilises a distance-based approach, with the rationale that non-uniform positioning of a compartment will be revealed by non-uniform distributions of experimental intercompartment distances compared with those calculated for a uniform simulated distribution [19]. This approach has been successfully applied to chromosome territories by basing homologue separation distances on the distances between the centres of homologous chromosome territories [19,20,33], satellite DNA sequences [28,34], genomic loci in Drosophila cell nuclei [11], and Barr bodies in human fibroblasts [35]. It is also possible to quantify relative spatial proximity in terms of orbital arrangement using the angle formed between compartments and the nuclear centre. This method was originally used to study the positions of chromosomes on prometaphase chromosome rosettes [19,36] and has been used more recently in interphase cells [20], where the wide range of angles measured between homologous chromosomes argued against the existence of a highly ordered orbital arrangement of chromosomes.

In the future it is likely to become common to use a combination of different criteria to give a detailed picture of how compartments are positioned in the nucleus. This more systemic approach was recently used to describe the tissue-specific spatial positioning of chromosomes [33] and in a global study by Bolzer and colleagues, in which all 46 chromosome territories were investigated simultaneously using distance-based radial/relative positioning in combination with angle-based relative positioning [20].

## Looking for Overlap

The nucleus is a crowded space, and it is not unusual for nuclear compartments to be observed close to one another or even overlapping. Whether such associations are occurring more often than might be expected by chance alone is an important issue in understanding nuclear organisation and one that can really only be addressed by quantitative means.

Association frequency is one example of a commonly used method to judge the degree of interaction between compartments. Association can be either loosely defined as compartments having adjacent or overlapping signals [37–42], or based more quantitatively on some degree of pixel overlap between fluorescent foci, where, for example, greater than 50% of the pixels may be required to overlap for a positive association to be recorded [15]. Often studies simply look for differences in the frequency of association, for example, by comparing the frequency of association of different genomic loci for the same nuclear compartment [40,43–45]. Otherwise, identification of preferential association may be achieved using theoretical models of random colocalisation, where the probability of colocalising is calculated based on total cell nucleus volume together with the size and number of the compartments of interest [10,46–48].

An alternative method is to use distance-based approaches, where distances are those measured between the centroids (or the intensity gravity centres) of the nuclear compartments of interest. This method is probably most applicable to those compartments having simple focal distribution patterns and has been particularly useful in quantitating the degree of physical proximity between genomic loci involved in imprinting [49], radiation-induced chromosomal rearrangements [50], and chromosome translocations [51,52]. It has also been used to quantitate relationships between genomic loci and a number of nuclear compartments, including PML nuclear bodies [53,54] and centromeres or telomeres [55]. As for association-based approaches, it is common to use differences in measured distances to compare the degree of interaction between genes and/or compartments. A more sophisticated version of this type of approach is to use cross-pair correlation, a statistical measure used to investigate correlations between two sets of point-like objects. Based on a histogram of interfocal distances, clustering of compartments is indicated if certain distances are found more often than expected. Conversely, if distances are found less often than expected, then the compartments show regularity [56]. The still limited resolution of optical microscopy, especially in the vertical direction, may prevent us from seeing close intergenic or interfocal distances, which could potentially bias any distance-based methods of analysis. However, the ability to improve the resolution via cryo-immunofluorescence [57], quantum dots [58], and the recently demonstrated ability to bypass the diffraction limit [59] means that these limitations may be short-lived.

Both association-based and distance-based approaches rely on the ability to identify and delineate nuclear compartments as discrete fluorescent foci. Colocalisation of fluorescent spectra is an alternative yet simple procedure to estimate the degree of association between nuclear compartments, with the advantage that segmentation is not necessarily required prior to analysis. Using this method, semi-quantitative colocalisation assessments can be made by plotting the signal intensities of the two fluorescent channels from a set of arbitrarily chosen lines drawn through the image. A positive colocalisation is indicated by coincidence in the signal intensity for the two fluorescent channels [60]. Comparison of fluorescent density profiles has been applied to a diverse range of nuclear compartments, including glucocortocoid receptor clusters [60], chromosome territories [61–63], and DNA helicase II foci [64]. A more quantitative method to study colocalisation based on pixel overlap, especially for nuclear compartments with very complex microspeckled distribution patterns, is the cross-correlation method [16,47,65–67]. In this method, two differentially labelled 3D images are shifted with respect to one another along the *x*- or *y*-axis, and the amount of pixel overlap is calculated for each shift. A decrease in the amount of overlap will result if the nuclear compartments are significantly colocalised in the original non-shifted image. Conversely, if the pixel overlap is minimal in the original image, then shifting the images over a small distance will lead to an increase in the amount of overlap. If the nuclear compartments are uniformly distributed with respect to one another, a small shift should have virtually no net effect on the amount of overlap. The validity of this method was tested by a computer simulation in which artificial 3D nuclear images were constructed containing 1,000 red and green spots of roughly the size of the experimental nuclear foci involved. Images were generated in which the red and green spots were either completely or partially overlapping, mutually excluding, or independently distributed [65]. These simulations demonstrated that the cross-correlation method was a powerful tool to distinguish positively or negatively correlated subnuclear distributions from unrelated distributions. A major advantage of cross-correlation analysis is that it does not require image segmentation techniques that separate objects from background. Such segmentation techniques are often susceptible to errors and generally depend on a priori assumptions regarding the size, shape, or labelling intensity of the objects in the image. A number of restrictions do however apply, in that the nuclear compartments should be small compared with the overall size of the image, they should be homogenous in size, and their shape should be isotropic rather than irregular [65].

It is worth emphasising that even when nuclear compartments are found to have statistically significant associations, the interpretation may not always be clear. The possibility exists that compartments having a preference to occupy the same nuclear space (e.g., the nuclear periphery) may be more likely to have high associations. Whether these associations can be definitively interpreted as evidence of a functional interaction is a matter for further debate. At the present time, no sophisticated nucleus-wide, function-defined model of compartment distributions exists, and therefore test procedures refer to simple null distributions. However, colocalisation studies can be effective, as demonstrated by McManus and colleagues [68], who explored the spatial relationship between CREB binding protein and other compartments and did not observe the expected relationships. By adopting a distance-based statistical probability model, it was shown that CREB binding protein has a higher than expected probability of being in spatial proximity to specific nuclear compartments, with no a priori knowledge of these associations other than biochemical evidence that would support such associations.

## Dealing with Large or Irregularly Shaped Compartments

Not all nuclear compartments are conveniently punctate or spherical in their intranuclear distribution pattern. Chromosome territories, nucleoli, and splicing speckles are all examples of compartments with complex distributions or irregular outlines. Methods to study such compartments in a statistically meaningful manner are more limited and are generally complicated by the inability to assign precise nuclear coordinates for their locations based on centroid position. Distance-based radial analysis methods are therefore not always applicable, or require some degree of approximation; for example, chromosome territory centroid position is often estimated as the intensity gravity centre. An alternative way to quantitatively analyse nuclear position is to measure the radial position of all voxels that define the compartment of interest (Figure 3) [20,23]. Association-based methods of analysis present more of a challenge, especially for compartments occupying a relatively large proportion of the nucleus. Splicing speckles, for example, have been estimated to occupy 5% of the total nuclear volume [69], and because of their convoluted surface, they have a large surface area with which other nuclear compartments can potentially associate or overlap. At present, pixel overlap or cross-correlation–based methods are the only quantitative means by which associations involving these types of complex compartments can be investigated.

The fact that nuclear compartments can occupy a relatively large proportion of the nuclear volume can also have consequences for the quantitative analysis of other unrelated nuclear compartments. This is because some nuclear compartments may form exclusion zones into which other compartments cannot gain entry; for instance, both chromosome territories and nucleoli have been reported to exclude certain other compartments [10,70]. In these cases, it is important to be able to subtract exclusion zone volume from overall nuclear volume. Estimating the volume of such exclusion zones requires calculating the volume of irregular domains, and this can be done most simply by applying the Cavalieri principle. This is a practical alternative to the Archimedes principle of estimating volume by water displacement: it estimates volume by adding together the areas of the object in serial cut sections. The Cavalieri principle is directly applicable to volume estimation of nuclear compartments using data generated by 3D microscopy [23,71,72]. For more accurate volumes, image segmentation methods such as the Voronoi tessellation procedure have to be applied [73]. Such procedures essentially subdivide the image into progressively smaller polygons until each polygon represents an area with similar pixel intensity. The shape of the nuclear compartment is then extracted from the Voronoi diagram by showing all polygons over a certain pixel threshold. The ability to calculate volumes and shapes is likely to become more important as we develop more sophisticated models of how the interior of the nucleus is organised. This is mainly because exclusion zones decrease the effective volume within which a nuclear compartment of interest may be distributed, and the larger the exclusion zone, the more impact it will have on the accuracy of the resulting model if it is not taken into consideration.

The ability to calculate compartment volume can have other uses. For example, chromosome territory volume can also be used to assess the degree of chromatin compaction and has proven useful in investigating differences in compaction relating to gene density [23]. Alternatively, measures of chromatin compaction can be based on distances between genomic loci [72,74] or replication foci within territories [75]. For an understanding of their internal structure, territories are often considered to have spherical or ellipsoid shapes, making statistical analysis of the spatial intraterritorial arrangement more convenient. Erosion methods, similar to that described previously for intranuclear radial analysis, can then be used to subdivide the territory volume into layers to investigate the intraterritory distribution of genomic sequences or nuclear compartments. Detailed studies on a range of genes and gene clusters by such methods have demonstrated that intraterritory organisation is non-uniform, that actively expressed chromatin can have preferred locations toward the interior, periphery, or exterior surface of the territory [12,45,74,76–78], and that replication foci are distributed throughout the territory volume [79]. These studies differ markedly in the methods used to define the location of the gene with respect to the fuzzy boundary of the chromosome territory, ranging from simple visual inspection of the data to more accurate representations of the territory border based on a range of thresholds.

## Conclusions

It is almost 20 years since Belmont and colleagues identified the need for methods to both rapidly acquire and analyse data on the spatial distribution of nuclear compartments in a statistically meaningful way [35]. We are now technically capable of acquiring extremely high resolution images of the cell nucleus and have a broad range of statistical methods with which to study these data. A systemic approach to how we study cell nuclear organisation is now required, one which would include commonality in the methods by which we visualise, process, and analyse cell nucleus data, thereby making the task of comparing and collating quantitative analyses between different laboratories much simpler.

The quantitative methods described here are remarkably flexible in their application, with radial positioning methods being useful to investigate both gene loci positioning within chromosome territories and chromosome territory positioning within the cell nucleus. Similarly, interpoint, distance-based approaches can be used to assess both whether a compartment is non-uniformly distributed in the nuclear space and whether it has statistically significant associations with other compartments. The choice of statistical method used will often depend on the type of nuclear compartment under investigation. Some compartments, especially those of spherical and focal configurations, are generally more straightforward to study since their nuclear location can easily be defined in terms of their centroid position. In fact, the similarity of many nuclear compartments to spatial point patterns encountered in other disciplines, such as statistical physics and epidemiology, is likely to lead to more sophisticated methods to study the cell nucleus in the near future, as demonstrated in recent publications [80,81]. Other compartments are simply not amenable to distance-based quantitative analysis, especially those of irregular outline, such as the splicing speckle compartment, or the interconnected mesh of foci formed by the inactive RNA polymerase II compartment. In these cases, novel classification methods not relying on segmentation and image thresholding would greatly increase our ability to identify and differentiate these more complex nuclear compartments.

Accurate quantitative description of nuclear compartment spatial organisation in static cells is only the first step toward an overall understanding of how the cell nucleus functions. It is not unlikely that seemingly independent nuclear processes may have unforeseen influences on one another. A combination of quantitative experimental data and in silico modelling will therefore be increasingly useful to simulate interactions between multiple nuclear processes (discussed more fully in [82]). These models will also have to cope with quantitative data describing the dynamic nature of the processes occurring inside the cell nucleus, as determined from our expanding use of in vivo microscopy to visualise the cell nucleus. The ultimate end point will be to have a fully functioning model of a virtual cell nucleus and to uncover the basic principles underlying its functional organisation. 

## Supporting Information

### Glossary

**centroid:** The geometric centre of a nuclear compartment; when the compartment is nonspherical (e.g., chromosome territories), the centre is usually taken as the intensity gravity centre.

**image segmentation:** Images are often segmented in order to separate out regions of the image corresponding to objects from those regions corresponding to background. Segmentation is determined by a single parameter known as the threshold value.

**pixels:** Every pixel in an image has a pixel value, or intensity, indicating how bright that pixel is. For a grayscale image the pixel value is a single number with a range of possible values between zero and 255, where zero is black and 255 is white. Values between zero and 255 make up the different shades of gray.

**radial analysis/peeling:** An approach to hypothesis testing that involves partitioning the nuclear volume into non-overlapping, contiguous regions. The test statistic compares the observed numbers of compartments in each region with the expected numbers under some null hypothesis, often complete spatial randomness.

**random (spatial) distribution:** The location of a compartment is random if it cannot be precisely specified prior to observing the nucleus. Common use is taken to imply that any location is equally likely (see “uniformity”).

**resolution:** The minimum distance measurable between two points.

**thresholding:** A specified pixel value is chosen as the threshold value. During thresholding, pixels in the image lower than the threshold value are set to zero (or black) and pixels higher than the threshold are set to 255 (or white). For colour images, different threshold values can be specified for red, green, and blue.

**uniformity:** The uniform distribution assigns the same probability to regions of equal size. In terms of the spatial distribution of compartments (with some technical assumptions), a uniform spatial distribution would be taken to mean complete spatial randomness.

**voxel:** A volume element; the 3D analogue of a pixel.

## Abbreviations

2D: two-dimensional
3D: three-dimensional
FISH: fluorescence in situ hybridisation
PML: promyelocytic leukaemia
